# Potential causal association between aspirin use and the reduced risk of hayfever or allergic rhinitis: a Mendelian randomization study

**DOI:** 10.3389/fimmu.2023.1232981

**Published:** 2023-08-25

**Authors:** Li Li, Yuanding Zhang, Xiangliang Liu, Junxin Li, Qiuyu Yang, Jiajia Jiang, Hong Liu, Zhongying Fu, Weilun Chen

**Affiliations:** ^1^ Department of Otolaryngology-Head and Neck Surgery, Lequn Branch, The First Hospital of Jilin University, Changchun, China; ^2^ Cancer Center, The First Hospital of Jilin University, Changchun, China; ^3^ Department of Rehabilitation Medicine, The Second Hospital of Jilin University, Changchun, China

**Keywords:** aspirin, allergic rhinitis, causal analysis, Mendelian randomization, single nucleotide polymorphism

## Abstract

**Background:**

The evidence from observational studies on the association between the use of aspirin and the risk of hayfever or allergic rhinitis is conflicting, with a dearth of high-quality randomized controlled trials.

**Objective:**

This study aims to investigate the causal relationship between aspirin use and the risk of hayfever or allergic rhinitis.

**Methods:**

We conducted a two-sample Mendelian randomization (MR) analysis using the inverse-variance weighted (IVW), weighted median, and MR-Egger regression methods. We utilized publicly available summary statistics datasets from genome-wide association studies (GWAS) meta-analyses on aspirin use in individuals of European descent (n = 337,159) as the exposure variable, and a GWAS on doctor-diagnosed hayfever or allergic rhinitis in individuals from the UK Biobank (n = 83,529) as the outcome variable.

**Results:**

We identified 7 single nucleotide polymorphisms (SNPs) at genome-wide significance from the GWASs associated with aspirin use as instrumental variables (P<5×10−8; linkage disequilibrium r2 <0.1). The IVW method provided evidence supporting a causal association between aspirin use and reduced risk of hayfever or allergic rhinitis (β = -0.349, SE = 0.1356, P = 0.01008). MR-Egger regression indicated no causal association between aspirin use and hayfever or allergic rhinitis (β = -0.3742, SE = 0.3809, P = 0.371), but the weighted median approach yielded evidence of a causal association (β = -0.4155, SE = 0.1657, P = 0.01216). Cochran’s Q test and the funnel plot indicated no evidence of heterogeneity and asymmetry, indicating no directional pleiotropy.

**Conclusion:**

The findings of the MR analysis support a potential causal relationship between aspirin use and the reduced risk of hayfever or allergic rhinitis.

## 1 Introduction

Hayfever or allergic rhinitis is an allergic condition characterized by symptoms triggered by allergen exposure, such as nasal itching, nasal congestion, sneezing, and a runny nose (1). Patients may also experience conjunctival allergies and pruritus. The conventional treatment for allergic rhinitis involves glucocorticoid nasal sprays, while antihistamines, leukotriene modifiers, or nasal irrigation can provide symptomatic relief for patients with mild symptoms. However, there is currently no medication available for long-term effective control of allergic rhinitis. Previous studies (2) have shown that long-term oral aspirin therapy can induce immune tolerance in certain patients with Aspirin-Exacerbated Respiratory Disease (AERD), leading to alleviation of respiratory symptoms. Nevertheless, limited research has explored whether aspirin use can reduce the risk of hayfever or allergic rhinitis.

In the context of aspirin use and allergic rhinitis, previous studies (3) have primarily focused on its effects in patients with Aspirin-Exacerbated Respiratory Disease (AERD). AERD is a condition where aspirin and other nonsteroidal anti-inflammatory drugs (NSAIDs) exacerbate respiratory symptoms in individuals with asthma and nasal polyps. It has been observed that long-term oral aspirin therapy can induce immune tolerance in some AERD patients, leading to improved respiratory symptoms. However, the potential impact of aspirin use on the risk of developing hayfever or allergic rhinitis has not been extensively investigated.

To address this research gap, the current study aims to apply a two-sample Mendelian randomization (MR) (4) approach to explore the potential causal relationship between aspirin use and the risk of allergic rhinitis. MR is a method that employs genetic variations as instrumental variables (IVs) to evaluate whether observed associations between risk factors and outcomes indicate a causal relationship (5). Genetic variants associated with aspirin use will be used as instrumental variables in one sample, while the outcome of allergic rhinitis will be measured in a separate sample. By leveraging large-scale genetic data and summary statistics from GWAS, this study can provide insights into whether there is a causal association between aspirin use and a reduced risk of allergic rhinitis.

Understanding the potential protective effects of aspirin against allergic rhinitis could have significant clinical implications. If the results of this study suggest a causal relationship, it could inform the development of novel prevention strategies or the repurposing of aspirin as a long-term treatment option for allergic rhinitis. However, it is important to note that the findings of this MR study should be interpreted in the context of its limitations, including the assumptions underlying the MR framework and potential pleiotropy of the genetic instruments. Further research, including randomized controlled trials (RCTs), will be needed to confirm and validate the results of this study.

## 2 Materials and methods

**Figure 1:**
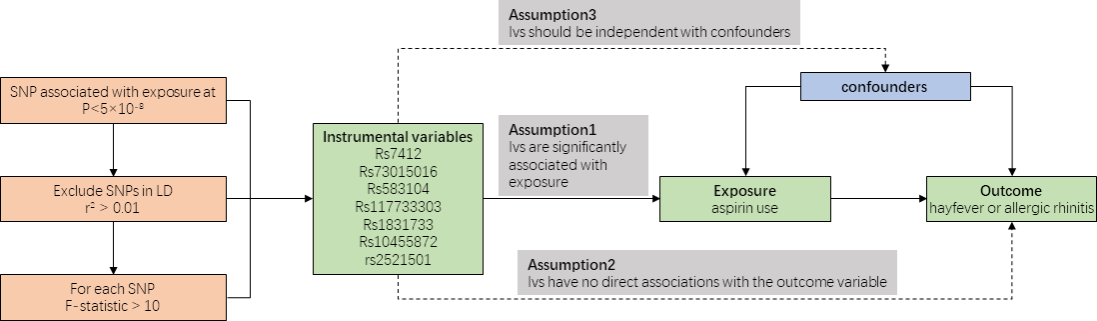
Overall design of Mendelian randomization analyses.

**Figure 1** provides an overview of our study design. As our data were derived from published studies and publicly available databases, we did not require additional ethical approval from an institutional review board.

### 2.1 Data sources and selection of genetic variants

We acquired summary statistics datasets from the MR Base database (6) and the UK Biobank, which are publicly available. The exposure variable in our analysis comprised the GWAS meta-analyses on aspirin use among individuals of European descent (n = 337,159), while the outcome variable consisted of the GWAS on doctor-diagnosed hayfever or allergic rhinitis among individuals from the UK Biobank (n = 83529). The detailed information of the GWASs is listed in **Table 1**.

**Table 1:** Details of studies and datasets used in the study.

Exposure/Outcomes	Web Source	ID	Sample Size	SNP Size	First Author	Consortium	Year	Population Studied
aspirin use	http://www.mrbase.org/	ukb-a-132	337159	10894596	Neale	Neale Lab	2017	European/Males and females
hayfever or allergic rhinitis	http://www.mrbase.org/	ukb-a-254	83529	10894596	Neale	Neale Lab	2017	Mixed/Males and females

GWAS, genome-wide association study; SNP, single nucleotide polymorphisms.

### 2.2 Strength test and selection of instrumental variables

Instrumental variables (IVs) are selected based on predetermined criteria. These IVs should exhibit strong correlations with the exposure variable and have no direct associations with confounding factors or the outcome variable. The selection of appropriate IVs helps establish a causal relationship between the exposure variable and the outcome variable, such as the influence of aspirin use on the occurrence of allergic rhinitis or hayfever.

In our study, we included seven SNPs that were associated with aspirin use as instrumental variables (IVs). Each SNP exhibited a robust correlation (P < 5 × 10^-8^) with the exposure variable, satisfying the predetermined criteria for instrumental variables. No linkage disequilibrium (LD) was found among the 7 instrumental SNPs (r^2^ threshold < 0.1). the F-statistics was calculated and all 7 SNPs satisfying the assumption of F >10 for MR analyses (7).

### 2.3 Estimation of the causal relationship between aspirin use and hayfever or allergic rhinitis

Mendelian randomization (MR) analysis relies on the assumptions of genetic stability and adherence to Mendel’s laws of inheritance, ensuring unbiased allocation of parental alleles to offspring and maintaining the directionality of causal inference (8). By leveraging GWAS, which utilize SNPs and population-level phenotypic data to assess statistical associations, we can examine the strength of the SNP-phenotype relationship and preserve the directionality of causal inference. We show the analysis process of Mendelian randomization in **Figure 1**.

To investigate the causal association between aspirin use and hayfever or allergic rhinitis, we employed three statistical methods: the inverse-variance weighted (IVW) method, the weighted median estimator, and MR-Egger regression. The IVW method conducts a meta-analysis of Wald ratio estimates for the included SNPs, producing weighted estimates to infer causal relationships (9). The weighted median estimator calculates the causal effect by sorting the effect estimates of individual SNPs based on their weights and taking the median value (10). This method requires a valid variable proportion of at least 50% to provide a consistent estimate of the causal effect while reducing the influence of invalid variables. MR-Egger regression can be used even when all SNPs are invalid. It estimates the causal effect through a weighted linear regression of gene-outcome coefficients on gene-exposure coefficients. The slope of MR-Egger regression represents the causal effect (11), while the intercept provides an estimate of the average horizontal pleiotropic effect across genetic variants. Causal relationships are determined based on statistical significance, with a p-value less than 0.05 indicating a significant causal relationship, suggesting that the observed differences are unlikely to be due to random variation but rather reflect the actual causal impact of the exposure on the outcome.

### 2.4 Sensitivity analysis

We assessed heterogeneity between SNPs by utilizing Cochran’s Q-statistics and I^2^ statistic. To explore the influence of individual SNPs on the causal association, we performed a “leave-one-out” analysis (12). This involved systematically removing each SNP one by one and recalculating the effect of the remaining SNPs using the IVW method. By doing so, we were able to examine the impact of each individual SNP on the causal inference and evaluate their contribution to the overall results.

## 3 Results

### 3.1 Detailed information of included SNPs

**Table 2** in the paper provides detailed information on each SNP, including its chromosome location, effect allele (EA), and effect allele frequency (EAF). The table also presents the associations of each SNP with Aspirin Use and Hayfever or Allergic Rhinitis, including the beta value, standard error (SE), and p-value. Among the SNPs analyzed, seven SNPs (rs7412, rs73015016, rs583104, rs117733303, rs1831733, rs10455872, rs2521501) demonstrated significant associations with both aspirin use and hayfever or allergic rhinitis. These SNPs exhibited statistically significant associations with both the exposure variable (aspirin use) and the outcome variable (hayfever or allergic rhinitis), indicating their potential role as instrumental variables (IVs). Additionally, the F statistics of these seven SNPs were found to be above 10, indicating their strength as IVs. F statistics measure the strength of instruments in Mendelian randomization analyses, with values above 10 generally considered robust instruments.

**Table 2:** Characteristics of the SNPs Associated with aspirin use and their associations with hayfever or allergic rhinitis.

SNPs	Chr	EA	EAF	aspirin use			hayfever or allergic rhinitis			F statistic
				β	SE	P	β	SE	P	
rs10455872	6:161010118	G	0.0810412	0.0133593	0.00150604	7.31E-19	-0.00682856	0.00376191	0.0694992	78.68497578
rs117733303	6:160922870	G	0.0186833	0.0196617	0.00303468	9.25E-11	-0.00807061	0.00759619	0.288032	41.97722735
rs1831733	9: 22076071	C	0.475704	0.00723124	0.000826446	2.15E-18	-0.00302929	0.00206174	0.141758	76.55860496
rs2521501	15: 91437388	T	0.322313	0.00490502	0.00088581	3.07E-08	-0.00294589	0.00221074	0.182687	30.66181305
rs583104	1: 109821307	G	0.42184	-0.00486719	0.00084236	7.56E-09	-0.0028417	0.00210676	0.177388	40.22202655
rs73015016	19: 11191300	T	0.773202	0.00622211	0.00098108	2.27E-10	-0.00137204	0.00245182	0.575751	32.78123454
rs7412	19: 45412079	A	0.118872	-0.00726876	0.00126954	1.03E-08	0.000856574	0.00317097	0.78706	38.51095695

SNPs, single-nucleotide polymorphisms; EA, effect allele; EAF, effect allele frequency; SE, standard error.

### 3.2 Mendelian randomization results

**Table 3** summarizes the results of the Mendelian randomization analysis. The IVW method revealed a positive association between aspirin use and the reduced risk of hayfever or allergic rhinitis, with an odds ratio (OR) of 0.7054 and a 95% confidence interval (CI) of 0.7084-0.5408. The weighted median estimator produced consistent results, showing an OR of 0.6600 with a 95% CI of 0.6600-0.4772. However, the MR-Egger method suggested a null causal effect.

**Table 3:** Causal associations between aspirin use and the reduced risk of hayfever or allergic rhinitis.

MR method	Number of SNPs	β	SE	OR (95% CI)	Association P‐value
MR Egger	7	-0.3742	0.3809	0.6878(0.3260-1.4512)	0.371
Weighted median	7	-0.4155	0.1655	0.6600(0.6600-0.4772)	0.0121
Inverse variance weighted	7	-0.349	0.1356	0.7054(0.7084-0.5408)	0.0101

CI, confidence interval; IVW, inverse variance weighted; OR, odds ratio; SE, standard error; SNPs, single-nucleotide polymorphisms.

**Figure 2:**
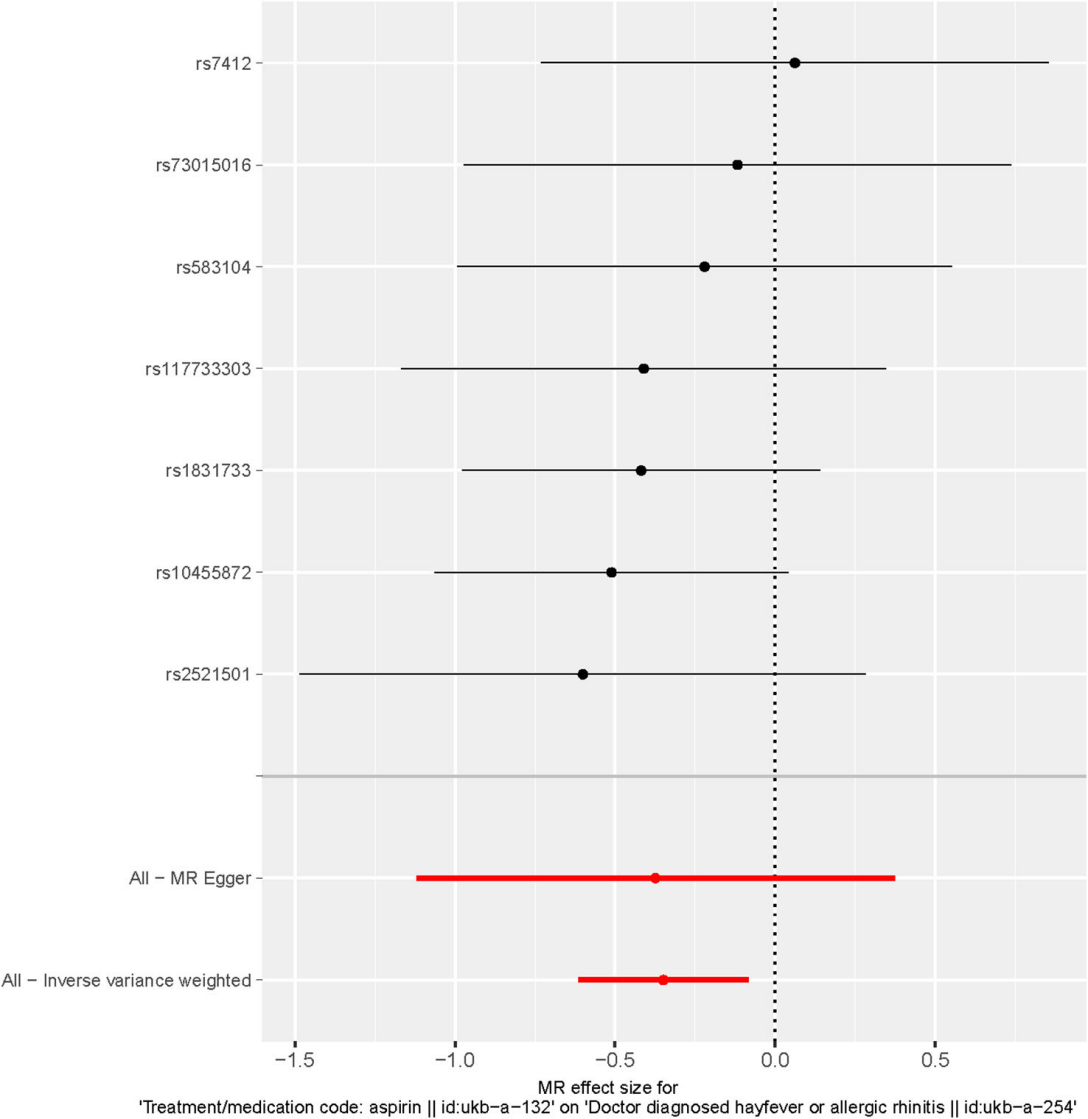
Forest plot of SNPs associated with aspirin use and hayfever or allergic rhinitis. Black points represent the log odds ratio (OR) for hayfever or allergic rhinitis per standard deviation (SD) increase in aspirin use, which is produced by using each SNP selected as a separate instrument (rs7412, rs73015016, rs583104, rs117733303, rs1831733, rs10455872, rs2521501). Red points show the combined causal estimate using all SNPs together as a single instrument, using the three different methods (the inverse variance weighted (IVW) method, weighted median estimator, and MR-Egger). Horizontal line segments denote 95% confidence intervals of the estimate.

**Figure 3:**
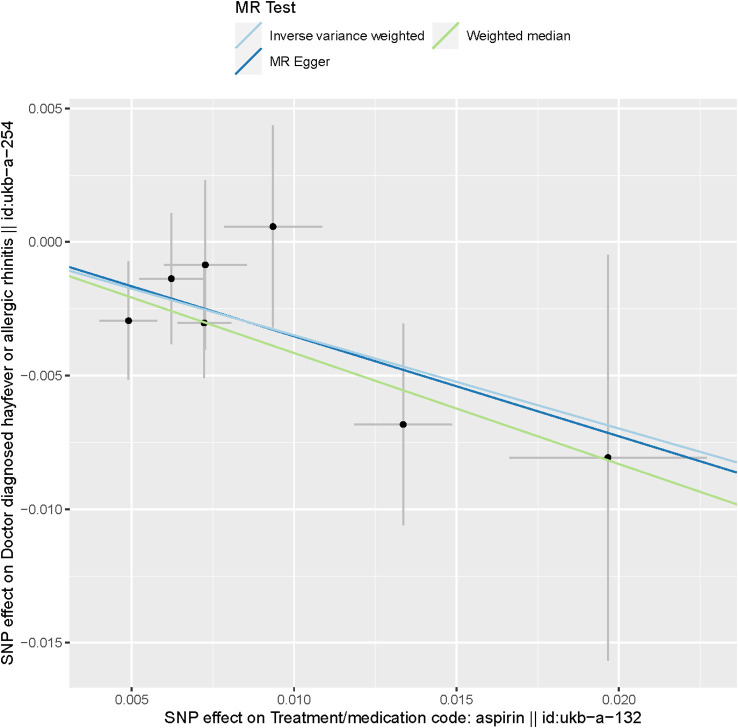
Scatter plot of SNPs associated with aspirin use and hayfever or allergic rhinitis. The plot presents the effect sizes of the SNP- aspirin use association (x-axis, SD units) and the SNP- hayfever or allergic rhinitis association (y-axis, log (OR)) with 95% confidence intervals. The regression slopes of the lines correspond to causal estimates using the three Mendelian randomization (MR) methods (the IVW method, weighted median estimator, and MR-Egger).

These findings are visually depicted in the forest plot (**Figure 2**) and the scatter diagram (**Figure 3**). The forest plot provides a graphical representation of the effect estimates and their confidence intervals for each SNP, while the scatter diagram illustrates the relationship between the exposure variable (aspirin use) and the outcome variable (hayfever or allergic rhinitis) using the instrumental variables.

Considering that the weighted median estimator yields more precise estimates compared to the MR-Egger analysis, the results of the Mendelian randomization analysis provide support for a potential causal association between aspirin use and the reduced risk of hayfever or allergic rhinitis.

### 3.3 Sensitivity analysis

**Figure 4:**
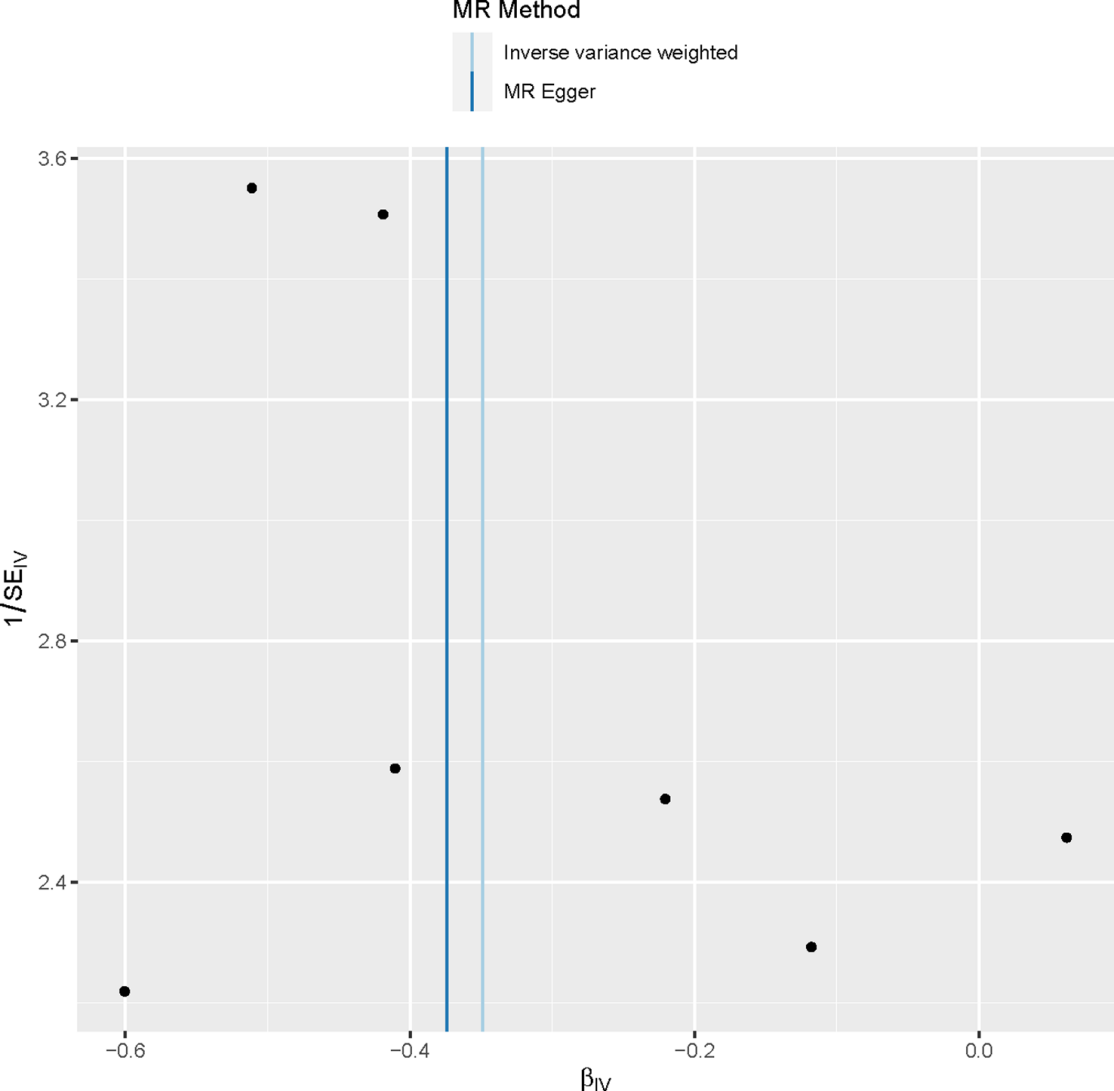
Funnel plot to assess heterogeneity. The blue line represents the inverse‐variance weighted estimate, and the dark blue line represents the Mendelian randomization‐Egger estimate.

The sensitivity analysis conducted in our study did not provide any evidence of genetic pleiotropy affecting the results. This is supported by the MR-Egger regression intercept, which was estimated to be 0.00021 (SE = 0.003, p = 0.946). Furthermore, the funnel plot and MR Egger regression test indicated no evidence of asymmetry, suggesting the absence of directional horizontal pleiotropy (**Table 4**; **Figure 4**).

**Table 4:** Sensitivity Analysis of MR. I^2^ = (Q − df)/Q.

MR method	Cochran Q statistic	I^2^	Heterogeneity P‐value
MR Egger	2.143	1.333	0.8291
Inverse variance weighted	2.148	1.793	0.9056

**Figure 5:**
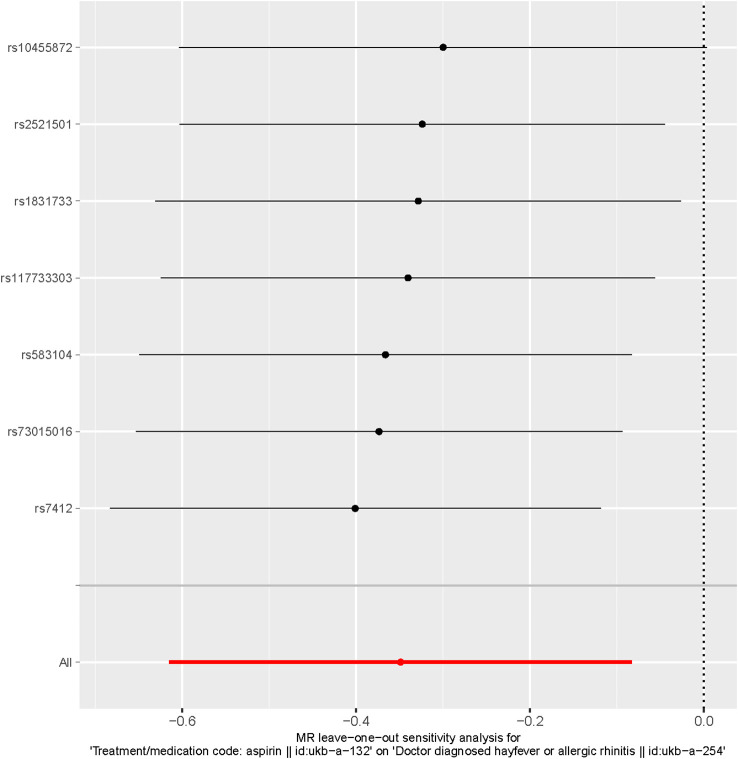
Leave-one-out of SNPs associated with Aspirin use and Hayfever or Allergic Rhinitis. Each black point represents result of the IVW MR method applied to estimate the causal effect of Aspirin use on the reduced risk of Hayfever or Allergic Rhinitis excluding particular SNP (rs7412, rs73015016, rs583104, rs117733303, rs1831733, rs10455872, rs2521501) from the analysis. Each red point depicts the IVW estimate using all SNPs. No single SNP is strongly driving the overall effect of Aspirin Use on the reduced risk of Hayfever or Allergic Rhinitis in this leave-one-out sensitivity analysis.

Additionally, the leave-one-out analysis demonstrated that no individual single nucleotide polymorphism (SNP) had a significant impact on the causal inference (**Figure 5**). This indicates that the overall causal association observed between aspirin use and the reduced risk of hayfever or allergic rhinitis was not driven by any specific SNP, highlighting the robustness of the findings.

These results provide reassurance regarding the validity and robustness of the causal inference established in the Mendelian randomization analysis.

## 4 Discussion

Allergic rhinitis, characterized by symptoms such as nasal itching, congestion, sneezing, and a runny nose, is a common allergic condition (13). It is caused by abnormal immune system reactions upon exposure to allergens like pollen, dust mites, or pet dander. The immune system responds with an excessive antibody response and releases inflammatory mediators, leading to nasal mucosa inflammation and allergic symptoms. Treatment options vary depending on symptom severity (14), ranging from antihistamines and nasal irrigation for mild symptoms to corticosteroid nasal sprays or oral antiallergic medications for more severe cases.

While previous studies have shown that aspirin use may trigger respiratory symptoms (15), the potential impact of aspirin use on allergic rhinitis occurrence has not been well-established through high-quality randomized controlled trials. Traditional epidemiological studies often face limitations in establishing causal relationships due to confounding factors. Therefore, the objective of this study was to investigate the relationship between aspirin use and the reduced risk of hayfever or allergic rhinitis using a two-sample Mendelian randomization study based on GWAS data.

In this study, we employed a two-sample Mendelian randomization approach to assess the potential causal relationship between aspirin use and a reduced risk of allergic rhinitis. We selected seven SNPs significantly associated with aspirin use as instrumental variables. By analyzing GWAS data for hayfever or allergic rhinitis using the inverse-variance weighted (IVW) method, weighted median estimator, and MR-Egger regression, we consistently observed a potential causal association between aspirin use and a reduced risk of hayfever or allergic rhinitis. These findings suggest that aspirin may have a preventive effect on the development of allergic rhinitis, providing new insights for further research on drug interventions for this condition.

It should be noted that aspirin use has been linked to the induction of respiratory-related symptoms. Aspirin-exacerbated respiratory disease (AERD) is characterized by the co-occurrence of asthma, chronic rhinosinusitis (CRS) with difficult to treat nasal polyposis, and intolerance to aspirin. The manifestation of aspirin intolerance entails the onset of acute symptoms that affect the upper and/or lower respiratory tract following the ingestion of aspirin. These symptoms are occasionally accompanied by acute dermatological rash or gastrointestinal manifestations. Similar symptoms can also be observed in some individuals who are exposed to other types of nonsteroidal anti-inflammatory drugs (NSAIDs), and this category of symptoms is collectively referred to as NSAID-exacerbated respiratory disease (NERD). Interestingly, some AERD patients may achieve aspirin desensitization by undergoing a period of low-dose aspirin intake, leading to alleviation of AERD-related symptoms. Similarly, some individuals with allergic rhinitis have reported symptom relief following aspirin intake (16). It is well-known that allergic rhinitis and AERD are two distinct diseases, with AERD not being induced by allergic reactions. However, they share certain similar symptoms, prompting us to consider whether there are similarities in the underlying mechanisms of these two conditions. Nevertheless, observational studies on this topic are prone to confounding factors, resulting in low-quality evidence. To date, high-quality studies explaining this phenomenon are lacking.

The exact mechanism by which aspirin use reduces the risk of allergic rhinitis remains uncertain. Allergic rhinitis is a non-infectious chronic inflammatory disorder mediated by IgE in the nasal mucosa (17). Upon allergen exposure, mast cells and basophils release inflammatory mediators, such as histamine, leukotrienes, and prostaglandins, triggering an immediate allergic response characterized by nasal itching, sneezing, and rhinorrhea. Aspirin, through non-selective inhibition of cyclooxygenase (COX), interferes with the metabolism of arachidonic acid, inhibiting prostaglandin synthesis in the body. Prostaglandins, as inflammatory mediators, can mediate allergic reactions through different receptors, leading to symptoms such as nasal congestion and rhinorrhea (18). Therefore, it is speculated that the ability of aspirin to inhibit prostaglandin synthesis may contribute to its potential in reducing the risk of allergic rhinitis. Additionally, long-term oral administration of aspirin in some aspirin-allergic patients may induce immune tolerance, thus reducing the risk of allergic rhinitis. However, further experimental validation is needed to confirm these specific mechanisms.

This study has several strengths, including the use of a two-sample Mendelian randomization design to control for confounding factors and minimize reverse causality concerns (19). Additionally, the utilization of published GWAS data and meta-analysis data provided a large sample size and valuable genetic variation information. However, there are limitations to consider. Due to the lack of publicly available baseline data for both the exposed and outcome groups, further detailed analysis pertaining to this causal relationship is limited with the current dataset. This restriction hinders a more nuanced exploration of the causal relationship using the existing data. Concerning the data on the exposed group, it should be noted that some elderly individuals may self-administer aspirin for various purposes without seeking medical consultation, and they may not disclose their medication status, potentially influencing the accuracy of data analysis. Regarding the data on the outcome group, an important consideration is that some individuals with mild allergic rhinitis may overlook their symptoms and refrain from seeking medical attention, leading to an overall bias in the outcome data towards moderate and severe allergic rhinitis populations, warranting increased vigilance in subsequent research. While the outcome group may have included a minority of patients with concurrent AR and AERD, their impact on the causal relationship is likely limited, considering the large sample size of the outcome group and the relatively low prevalence of individuals experiencing both conditions simultaneously in real-life settings. Simultaneously, verifying genetic polymorphisms can be challenging, and despite using the MR-Egger method, potential misclassification of genetic polymorphisms cannot be completely eliminated. Furthermore, the GWAS data for aspirin use were based on European populations, while the hayfever or allergic rhinitis data originated from a mixed population. This introduces the possibility of population stratification bias, and the generalizability of the results to other populations remains uncertain, requiring further investigation. Lastly, over-identification issues in two-sample Mendelian randomization studies can lead to an overestimation of the association between SNPs and exposure. Subsequent prospective randomized controlled trials can provide further insights and validation of the conclusions reached in this study.

After thoroughly discussing and understanding the aforementioned issues, we can consider the potential value of this study in the prevention and treatment of allergic rhinitis. For example, for patients with severe seasonal allergic rhinitis, in addition to prophylactic use of glucocorticoids and antihistamines before the allergy season, it may be worth considering the prophylactic use of aspirin to alleviate severe nasal allergic reactions. For individuals with severe allergic rhinitis who have developed resistance to glucocorticoids, the addition of aspirin as a means of controlling allergic rhinitis symptoms could be considered. Through further research on the mechanism of action of aspirin, it may be possible to develop a medication that avoids the side effects of aspirin while utilizing a novel mechanism to assist in the treatment of allergic rhinitis. In order to obtain more accurate conclusions, further randomized controlled trials (RCTs) are necessary. However, due to the antiplatelet effects of aspirin and its potential to cause gastrointestinal haemorrhage, caution should be exercised in considering the inclusion of patients with severe gastrointestinal disorders in future research.

## 5 Conclusion

our study provides evidence supporting a potential causal association between aspirin use and a reduced risk of hayfever or allergic rhinitis. These findings contribute to the understanding of the underlying mechanisms involved in the development of allergic rhinitis. However, additional research is necessary to elucidate the specific mechanisms through which aspirin exerts its protective effect. Given the potential impact of aspirin on blood clotting function and its association with gastrointestinal ulcers or haemorrhage, high-quality clinical studies and evidence are required before considering its application in the prevention and treatment of allergic rhinitis within a certain scope.

## Data availability statement

The datasets used for the Mendelian randomization analyses in this study are publicly available. Researchers can obtain access to these datasets by making reasonable requests. The original contributions presented in the study are included in the article/supplementary material. Further inquiries can be directed to the corresponding author.

## Ethics statement

As the data used in this study were obtained from published studies and public databases, no additional ethical approval from an institutional review board was required. The patients or participants included in the original studies have previously provided their written informed consent to participate in those studies.

## Author contributions

LL conducted the data analysis and drafted the manuscript, while YZ participated in mining the database and drafting the manuscript. XL reviewed and proofread the manuscript. JL and QY contributed to the literature search and data analysis. JJ, HL, and ZF were responsible for creating the tables and figures of the results. WC designed the study, revised the manuscript, and provided technical support. All authors have made significant contributions to the article and have approved the final version for submission. All authors contributed to the article and approved the submitted version.
